# Symptom Laterality Concordance: Aortic Dissection Masquerading as Ipsilateral Lumbar Radiculopathy

**DOI:** 10.1007/s43465-026-01757-7

**Published:** 2026-03-06

**Authors:** Dingyuan Jiang, Jinglan You, Zhimin Hu, Xu Zhang, Qing Zhou, Lin Cheng

**Affiliations:** 1 Department of Spinal Surgery, Zhuzhou 331 Hospital, Zhuzhou, 412000 China; 2 Department of Science and Education, Zhuzhou 331 Hospital, Zhuzhou, 412000 China; 3 Department of Critical Care Medicine, Zhuzhou 331 Hospital, Zhuzhou, 412000 China; 4 Department of Ultrasound Medicine, Zhuzhou 331 Hospital, Zhuzhou, 412000 China; 5 Department of Interventional Radiography, Zhuzhou 331 Hospital, Zhuzhou, 412000 China; 6 Department of Orthopedics, Zhuzhou 331 Hospital, Zhuzhou, 412000 China

**Keywords:** Aortic dissection, Lumbar disk herniation, Misdiagnosis, Lower extremity ischemia, Case report

## Abstract

**Introduction:**

Aortic dissection is a lethal vascular emergency that may present atypically as isolated limb ischemia. A specific diagnostic pitfall, termed “symptom laterality concordance,” arises when these ischemic symptoms align with a preexisting, ipsilateral lumbar spinal pathology, creating a high risk of misdiagnosis.

**Case Report:**

We present a 64-year-old male with known right L4/5 lumbar disk herniation, initially diagnosed with radicular exacerbation due to right lower limb pain and sensory loss. The examination revealed right L5 radicular signs alongside a cool limb with a weakly palpable pulse. Vascular ultrasound showed right common iliac artery occlusion. Subsequent tearing chest pain led to a CTA diagnosis of Stanford type B aortic dissection. Despite emergent stenting, the patient succumbed to aortic rupture.

**Conclusion:**

This case highlights “symptom laterality concordance” as a critical cognitive trap, where a vascular catastrophe mimics an ipsilateral spinal condition. It underscores the need for clinicians to carefully consider vascular pathologies in all unilateral limb presentations. We advocate for a dual vascular–neurological assessment and propose a clinical algorithm to mitigate this risk.

## Introduction

Lumbar disk herniation (LDH) is among the most common diagnoses in spinal practice, typically presenting with low back pain and radicular symptoms [1]. However, its clinical presentation is highly variable, and the absence of classic features can complicate and delay diagnosis [2, 3]. In contrast, aortic dissection is a lethal vascular emergency that classically presents with sudden, severe tearing pain [4, 5]. Although uncommon, it can also manifest as isolated unilateral limb ischemia due to iliac artery involvement—a scenario requiring heightened clinical suspicion [6–8]. The diagnostic challenge is particularly acute in patients with known ipsilateral LDH, in whom new limb symptoms may be erroneously attributed to spinal pathology, overlooking a potentially fatal aortic dissection. This report illustrates this diagnostic pitfall and emphasizes the necessity of rigorous clinical evaluation to prevent adverse outcomes.

## Case Description

**Fig. 1:**
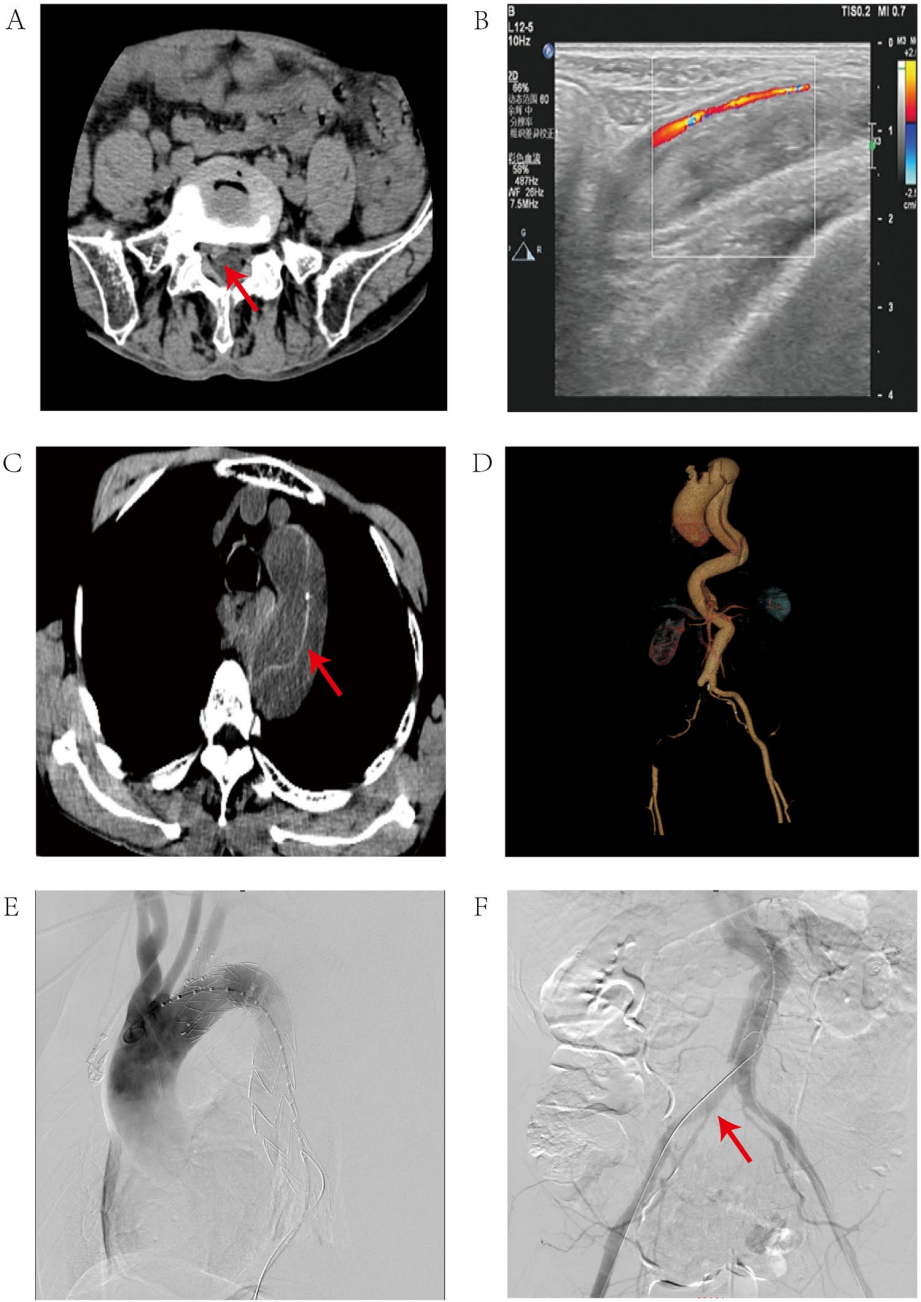
**A** Axial CT showing right posterior nucleus pulposus protrusion (arrow). **B** Vascular ultrasound demonstrating near-complete right common iliac artery occlusion (white box). **C** Axial CTA revealing aortic dissection flap (arrow). **D** 3D CTA reconstruction showing dissection extent and occluded right iliac artery (arrow). **E** Angiogram poststent placement. **F** Poststenting angiogram confirming iliac artery patency (arrow)

A 64-year-old male patient was transferred to our tertiary care center with a chief complaint of isolated, progressive right lower extremity pain and sensory disturbance refractory to oral analgesics. He had a documented history of right-sided L4/5 lumbar disk herniation, previously confirmed by computed tomography (CT) imaging (Fig. 1A). Upon admission, a detailed neurological examination was performed. Objective findings were consistent with right L5 nerve root involvement, including weakness in ankle dorsiflexion (tibialis anterior, grade 3/5) and great toe extension (extensor hallucis longus, grade 4/5), preserved plantar flexion strength, hypoesthesia to pinprick within the L5 dermatome (lateral calf and dorsum of foot), a diminished right Achilles reflex, and a positive straight leg raise test at 60 degrees reproducing radicular pain. Concurrently, the general physical examination revealed clear signs of acute right lower limb ischemia: the limb was cool and pale, the dorsalis pedis pulse was barely palpable, and the ankle-brachial index (ABI) was unrecordable, contrasting sharply with the normal left side (ABI 1.0). The striking concordance between these classic, ipsilateral radicular signs and the acute ischemic symptoms created a compelling but misleading clinical picture, initially suggestive of a radiculopathy exacerbation. Given the vascular compromise, an emergency lower limb duplex ultrasound was performed, demonstrating near-complete (≈90%) occlusion of the right common iliac artery (Fig. 1B). Several hours later, the patient’s condition deteriorated abruptly with nausea, vomiting, and severe tearing chest and upper abdominal pain. Immediate computed tomography angiography (CTA) diagnosed a Stanford type B aortic dissection (Figs. 1C, D). Emergent endovascular stent placement was performed (Fig. 1E), successfully restoring flow in the occluded right iliac artery (Fig. 1F). Despite intervention, he developed refractory hypotensive shock the following morning and succumbed, with the clinical course consistent with fatal aortic rupture and massive hemothorax. Written informed consent for publication was obtained from the patient’s next of kin.

## Discussion

This case elucidates a critical yet under-recognized diagnostic trap: “symptom laterality concordance.” This phenomenon occurs when the ischemic symptoms of a life-threatening vascular emergency, such as aortic dissection, present on the same side as a patient’s known degenerative spinal pathology (e.g., lumbar disk herniation), leading to cognitive anchoring and catastrophic diagnostic delay [1, 9].

The clinical presentation in this patient was a classic enactment of this pitfall. The detailed neurological examination revealed highly localizing signs of right L5 radiculopathy, perfectly aligning with his preexisting right L4/5 disk herniation and creating a “textbook” picture of spinal pathology. Simultaneously, unequivocal signs of acute right lower limb ischemia were present. The profound spatial overlap of these ipsilateral signs from two distinct disease processes generated a powerfully misleading clinical facade, triggering confirmation bias where new vascular symptoms were erroneously attributed to an exacerbation of the known spinal condition [3, 10].

Aortic dissection has a complex and heterogeneous presentation, with a reported misdiagnosis rate as high as 33.8% [10]. While classic tearing pain is a hallmark, up to 30% of cases may lack this symptom, and isolated limb ischemia is a recognized but easily missed initial manifestation [11, 12]. The key contribution of this case is its systematic illustration of how “symptom laterality concordance” uniquely amplifies this diagnostic challenge. It is not merely the coexistence of two pathologies, but an active cognitive trap that misdirects clinical reasoning. Although the pathophysiology of limb ischemia in dissection (branch vessel compression by the false lumen) is described [13], and case reports of limb ischemia exist [14, 15], the specific diagnostic bias induced by ipsilateral symptomatic overlap has not been adequately emphasized in the literature.

Based on this critical insight, we advocate for a reinforced assessment framework for unilateral limb symptoms. First, a basic vascular examination (palpation of bilateral pulses, assessment of skin temperature and color, and ABI measurement) must be a mandatory component of the initial evaluation for any suspected radiculopathy. Second, “symptom laterality concordance” should be explicitly recognized as an independent red flag warranting immediate vascular investigation. Third, noninvasive lower limb vascular ultrasound, a highly sensitive and specific tool, should be employed as a key first-line screening modality to compensate for the limitations of physical exam alone [16].

**Fig. 2:**
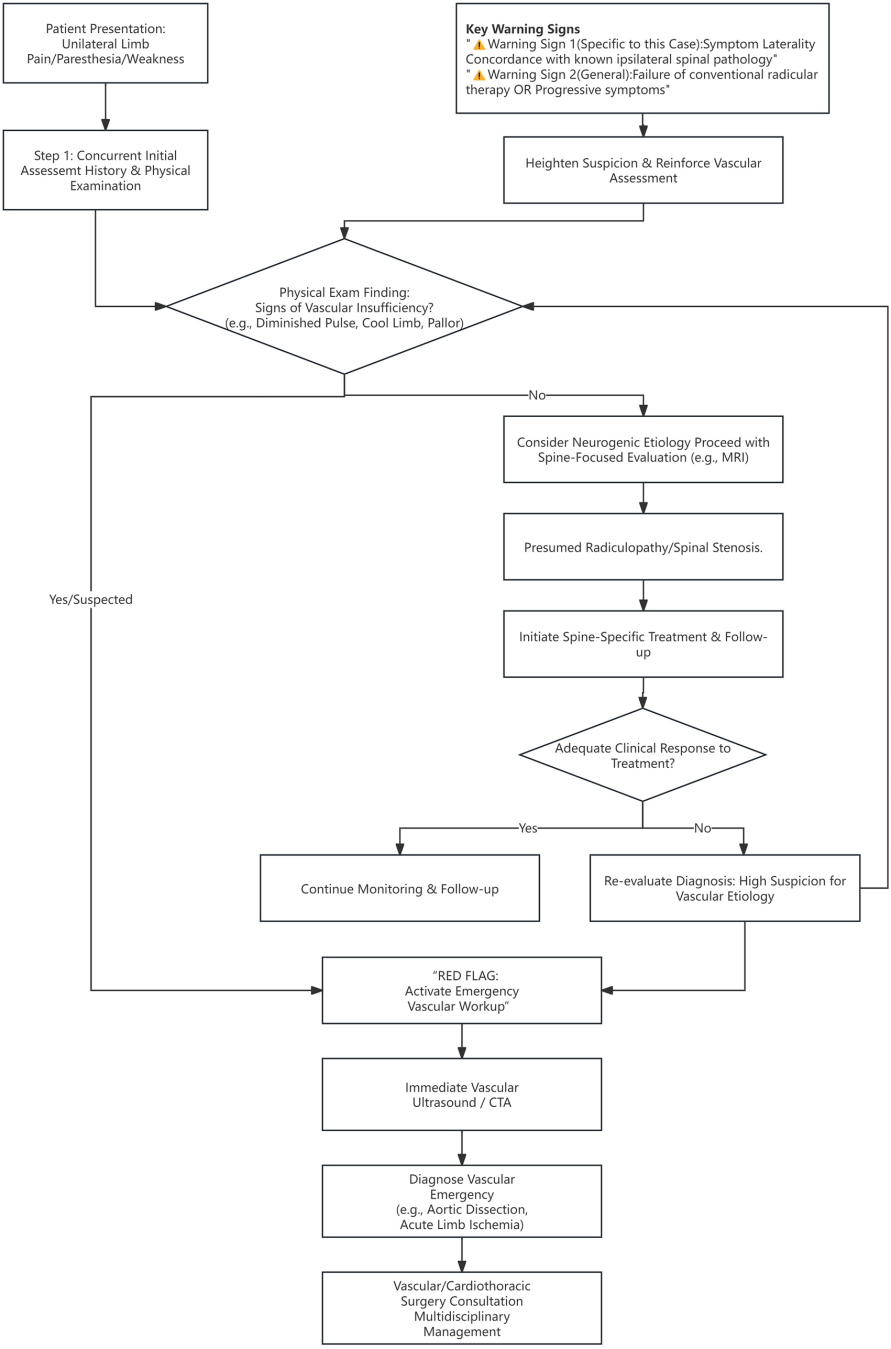
Proposed Integrated Assessment Flowchart for Unilateral Lower Limb Symptoms. Key features include: (1) the integration of specific warning signs, particularly symptom concordance with a known ipsilateral spinal condition, which should trigger immediate vascular investigation; (2) a safety feedback loop prompting reevaluation for vascular pathology if initial treatment for a presumed radiculopathy fails. *CTA* computed tomography angiography, *MRI* magnetic resonance imaging

To translate these recommendations into a tangible clinical tool, we have developed a structured assessment algorithm (Fig. 2). This algorithm outlines a “vascular-neurological dual consideration” approach, emphasizing the importance of parallel evaluation from the outset. A key element is the recognition of “symptom laterality concordance” as a high-risk diagnostic pitfall that should prompt immediate vascular investigation. Furthermore, it incorporates a safety feedback loop for cases where treatment for presumed radiculopathy fails, ensuring persistent symptoms prompt reevaluation for vascular causes. This algorithm may serve as a useful cognitive aid to help prevent similar diagnostic delays.

## Conclusion

“Symptom laterality concordance” represents a high-risk diagnostic blind spot in orthopedic, spinal, and primary care practice. This case serves as a reminder that in any patient presenting with unilateral limb symptoms, a definitive vascular etiology such as aortic dissection should be carefully considered—regardless of how compelling the evidence for a coinciding spinal pathology may be. Heightened awareness of this cognitive trap and the adoption of a systematic, dual-assessment approach may help avert similar tragedies.

## Data Availability

The data that support the findings of this study are available upon request from the corresponding author.

## Abbreviations

CT: Computer tomography
MRI: Magnetic resonance imaging
LDH: Lumbar disk herniation
CTA: Computed tomography angiography
3D: Three-dimensional
ABI: Ankle-brachial index
